# Supplementary material for: PG-path: Modeling and personalizing pharmacogenomics-based pathways
Source: PLoS One. 2020 May 4;15(5):e0230950. doi: 10.1371/journal.pone.0230950 (PMC7197763; doi:10.1371/journal.pone.0230950)
Supplement: S1 Table — (DOCX) [file pone.0230950.s001.docx]

**S1 Table. Interaction types and action types on Clopidogrel from DrugBank 5.0.1.**

| **Drugname** | **Uniprot_name** | **Gene** | **Interaction_type** | **Action** |
| --- | --- | --- | --- | --- |
| Clopidogrel | P2Y purinoceptor 12 | P2RY12 | target | antagonist |
|  | Cytochrome P450 3A4 | CYP3A4 | enzyme | substrate |
|  | Cytochrome P450 2B6 | CYP2B6 | enzyme | substrate, inhibitor |
|  | Cytochrome P450 3A5 | CYP3A5 | enzyme | substrate |
|  | Cytochrome P450 2C19 | CYP2C19 | enzyme | substrate |
|  | Cytochrome P450 2C9 | CYP2C9 | enzyme | substrate, inhibitor |
|  | Cytochrome P450 1A2 | CYP1A2 | enzyme | substrate |
|  | Cytochrome P450 2C8 | CYP2C8 | enzyme | inhibitor |
|  | Liver carboxylesterase 1 | CES1 | enzyme | substrate |
|  | Multidrug resistance protein 1 | ABCB1 | transporter | substrate |

Interaction type: the protein role, according to which the reaction between a drug and a gene happens; Action type: the type by which a drug acts to a protein or vice versa.
