# Supplementary figures and images for: PG-path: Modeling and personalizing pharmacogenomics-based pathways
Source: PLoS One. 2020 May 4;15(5):e0230950. doi: 10.1371/journal.pone.0230950 (PMC7197763; doi:10.1371/journal.pone.0230950)

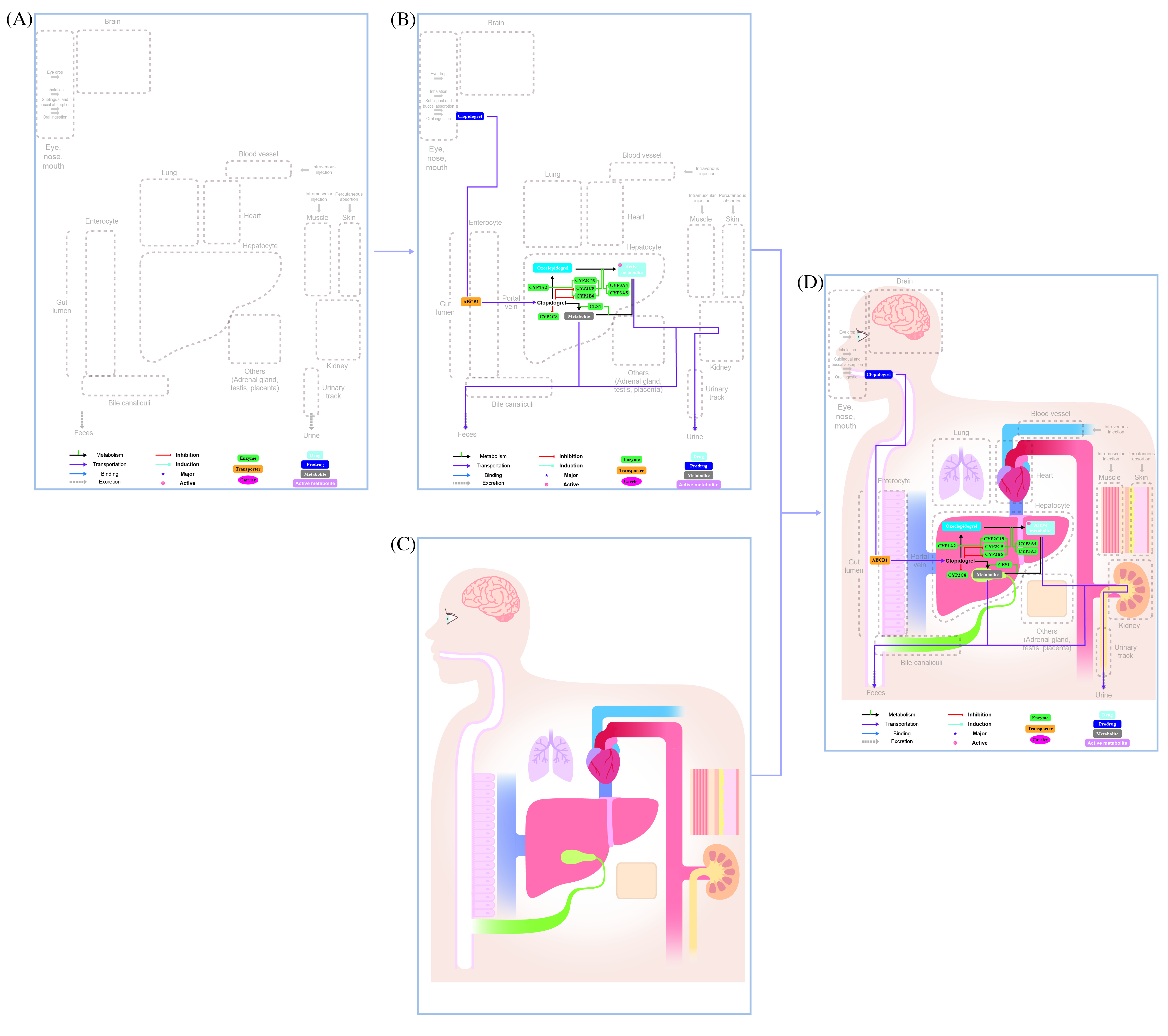

Supplement: S1 Fig — (TIF) [file pone.0230950.s002.tif]

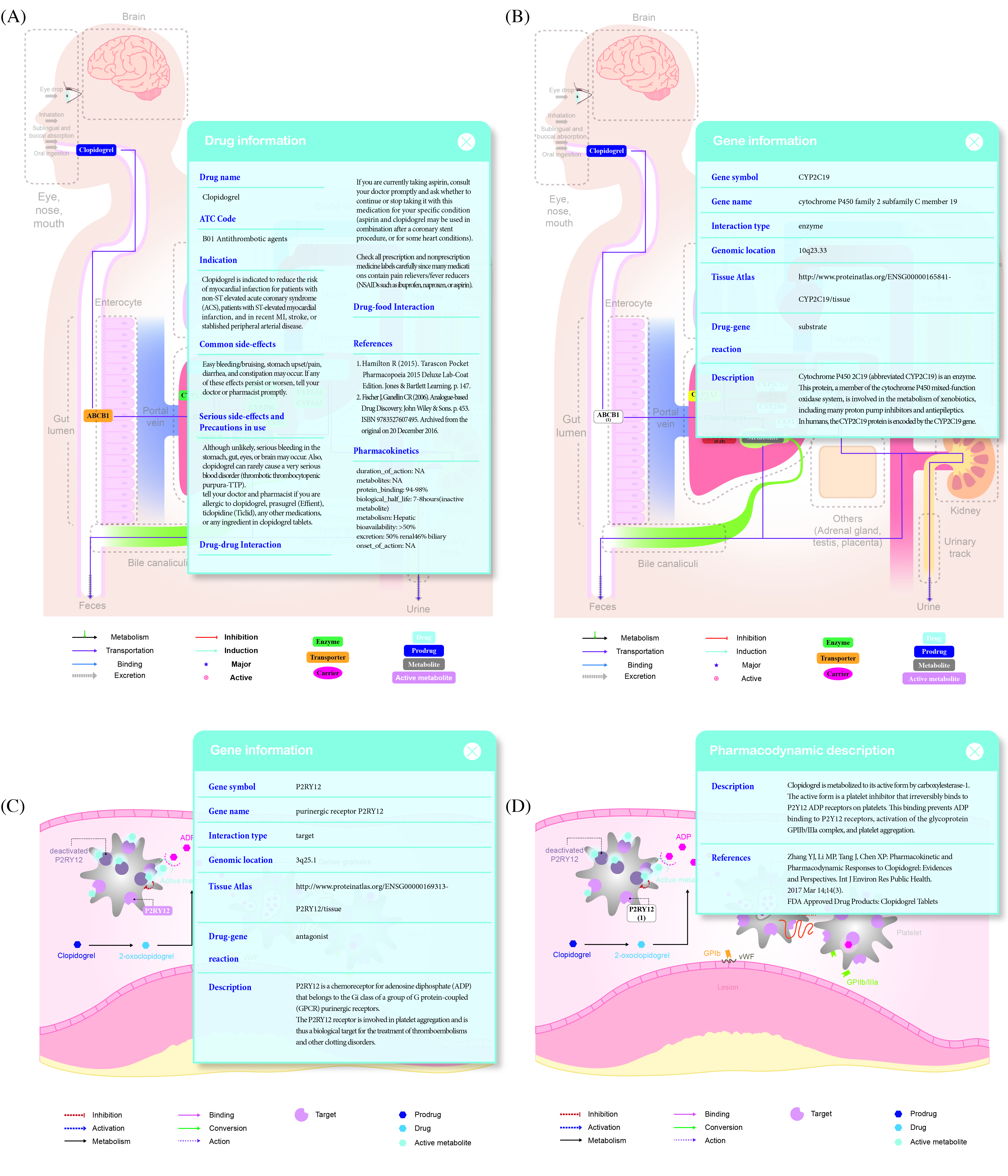

Supplement: S2 Fig — (TIF) [file pone.0230950.s003.tif]
