# Supplementary material for: PG-path: Modeling and personalizing pharmacogenomics-based pathways
Source: PLoS One. 2020 May 4;15(5):e0230950. doi: 10.1371/journal.pone.0230950 (PMC7197763; doi:10.1371/journal.pone.0230950)
Supplement: S1 File — (DOCX) [file pone.0230950.s004.docx]

$$S_{v}:nonsynanymous variant score$$

$S_{g}:Gene-wise Variant Burden$

$G_{j}:A set of variants in gene j$

*VCF: Variant call format*

$$VCF\mathrm{file}$$

Personal Genome Variation

Variant Score

$$S_{v_{j}}=SIFT score(v_{i})$$

$$S_{g_{j}}=\left( \prod_{v_{i}\in G_{j}}^{\infty} S_{v_{i}} \right)^{1/|G_{j}|}$$

Gene-wise Variant Burden Score
