# Supplementary material for: PG-path: Modeling and personalizing pharmacogenomics-based pathways
Source: PLoS One. 2020 May 4;15(5):e0230950. doi: 10.1371/journal.pone.0230950 (PMC7197763; doi:10.1371/journal.pone.0230950)
Supplement: Attachment — Submitted filename: Response to Reviewer.docx [file pone.0230950.s005.docx]

**Reviewer #1:**

1) It is not clear to this reviewer if PG-path will be a commercially available tool or a free downloadable one. Please clarify.

The objective of this study is modeling the pharmacogenomic pathway, so we do not determine yet whether PG-path will be available commercially or freely.

2) PG-path was designed based on available gene-drug information, and on data from the 1000 genome project. How can this pathway be continuously updated with the growing body of information that NGS technology provides every day?

We are currently working on the best methodology to incorporate the updates on time, though the detailed process will take time to determine.

3) Variants of the gene sequence are not the only ones that can impair the function and the activity of proteins involved in PK and PD processes. Is there any way to implement the path with epigenetic modifications and gene expression levels?

Epigenetic modifications are measured using chromatin immunoprecipitation (ChIP) and bisulfite-based methods, ChIP-chip and ChIP-seq, or RNA-seq. Gene expression is analyzed using microarray or RNA-seq. With our platform, the pathway is analyzed using DNA-seq. Therefore, it is not currently possible to apply epigenetic modification and gene expression level to PG-path. However, we will think carefully about these factors.

**Reviewer #2:**

1) It is not clear how users can access these new personalized pathways, applying the method described in the “Materials and Methods” paragraph. Is there a new tool, or is it being planned, to develop PK / PD pathways?

This paper only covers the steps of proposing a method for producing individual pathways. If the number of pathways increases, we are considering the creation of a website that can show results on the screen by searching distinct pathways in HTML format.

2) The authors used SIFT to predict the effects of genetic variants. Since we talk about predictions without any support coming from functional studies, having only one predictive program could be limiting. Other predictive programs should also be evaluated, for example, VarSome, an analysis tool for human genetic variation that reports prediction scores from 20 different algorithms.

In this study, pathway personalization was performed using the GVB score, defined as the geometric mean of the SIFT scores for the set of coding variants in a gene. However, evaluating the GVB score itself by applying various predictive scoring algorithms to GVB is an enormous task and should be conducted separately from this study.

3) The authors affirmed that this approach “addresses only genetic elements”. It would be advisable to speculate on this limit.

As mentioned, changes in drug concentration are influenced not only by genetic factors but also by clinical and environmental factors. These various factors will be investigated through further research.

4) The use of “gene deleteriousness” is inappropriate. It is more correct to state that the genetic variants are deleterious.

We have revised the term as you advise.
